# Editorial for the Special Issue “Vitamin K in Chronic Disease and Human Health”

**DOI:** 10.3390/nu14132595

**Published:** 2022-06-23

**Authors:** Evangelia Dounousi, Vasillios Liakopoulos

**Affiliations:** 1 Department of Nephrology, Faculty of Medicine, School of Health Sciences, University of Ioannina, 45110 Ioannina, Greece; 2 Section of Nephrology and Hypertension, First Department of Medicine, AHEPA Hospital, Medical School, Aristotle University of Thessaloniki, 54636 Thessaloniki, Greece; vliak@auth.gr

## 1. Introduction

Vitamin K and its derivatives represent a complex of fat-soluble vitamins, playing a major role in the regulation of a large number of physiologic processes required for optimal homeostasis [1]. Vitamin K was first described as a key factor implicated in blood coagulation nearly a century ago; however, accumulating evidence indicates pleiotropic actions of vitamin K extending well beyond the coagulation cascade [2,3]. Two main natural forms of vitamin K have been described, which differ in terms of not only structure but also kinetics of absorption and tissue distribution, bioavailability, and functional properties [3]. Vitamin K1 is mainly found in green vegetables (phylloquinone) and is also produced as a synthetic form (Phytonadione), whereas vitamin K2 (menaquinone) includes several forms characterized by an isoprenoid side chain of various lengths [1,3]. Meat and dairy products contain Vitamin K2 compounds, and they may also be generated from phylloquinone in the body or synthesized by the gut microbiota [1,3]. Despite the wide availability of vitamin K, a number of challenges remain in determining vitamin K status or body stores, especially with regard to vitamin K2.

The pivotal function of both vitamin K1 and K2 involves the hydroquinone form, serving as a cofactor for the enzyme gamma-glutamylcarboxylase, which catalyzes the generation of gamma-carboxyglutamic acid (Gla) residues in vitamin K-dependent proteins. During this process, hydroquinone is oxidized to vitamin K-epoxide, which is subsequently converted to hydroquinone by the vitamin K-oxidoreductase (VKOR), thus maintaining and perpetuating the vitamin K cycle [4,5].

Among the already identified vitamin K-dependent proteins, the coagulation cascade proteins, including factors VII, IX, X, and prothrombin, together with the regulatory proteins C and S, are the most well-acknowledged [4,5]. In addition, the spectrum of vitamin K-dependent proteins encompasses several proteins involved in the regulation of bone metabolism and vascular remodeling, such as matrix Gla protein (MGP), osteocalcin, and Gla-rich protein (GRP). Their main properties include the regulation of bone mineral deposition, the transition of osteoblasts to osteocytes, the inhibition of osteoclastogenesis, and the prevention of the development of ectopic calcifications in vascular smooth muscle cells and the extracellular matrix of blood vessels. Remarkably, recent research has brought to the forefront additional beneficial properties of vitamin K and specifically vitamin K2, including antioxidative and immunoregulatory effects, associated with the protection of the cellular membrane lipids from peroxidation and suppressed T-cell activation and proliferation. The beneficial physiological roles of vitamin K appear to translate into improved clinical outcomes in various organ systems, including the brain, the liver, the kidneys, and the cardiovascular system, among others [5].

However, despite the growing body of evidence on molecular mechanisms underlying the emerging new roles for vitamin K, there are several pending issues remaining to be elucidated by ongoing clinical research.

## 2. The Spectrum of Vitamin K and Related Markers—From Bench to Bedside

In light of the great body of evidence described above, the aim of this Special Issue is to present the latest evidence both tackling specifically the coagulation and extrahepatic activity of vitamin K as well as creating a frame by combining available data in order to further shed light on clinical implications of vitamin K and its derivatives.

Accordingly, two clinical studies published in this issue focus on critically ill patients with prolonged prothrombin times and examine the effects of vitamin K1 administration on the activity of vitamin K-dependent clotting factors and thrombin generation, and on vitamin K-dependent proteins unrelated to coagulation. Specifically, the results of the study authored by Dahlberg and colleagues showed that the coagulation status as measured by various assays improved overall in response to vitamin K1 administration in non-bleeding critically ill patients [6]. Interestingly, significant decrease in desphospho-uncarboxylated matrix Gla protein levels (dp-ucMGP) in response to vitamin K1 was observed in these patients. It should be noted that increased levels of -ucMGP have been associated with adverse outcomes in cardiovascular disease or diabetic chronic kidney disease [1,3]. On the other hand, findings from the study by Schött and coworkers indicate an association between vitamin K1 administration and increased levels of the growth arrest-specific gene 6 (Gas6) protein, a member of the extrahepatic vitamin K-dependent proteins [7]. Considering the already known pro-coagulant and anti-apoptotic properties of Gas6 linking it to thromboembolism and tumor growth, as well as the anti-cancer effects of vitamin K2, the question arises whether there are potential risks specifically associated with vitamin K1 treatment, and differences between K1 and K2 should be further clarified.

Rapp et al. performed a post hoc analysis on data of four different cohort studies including patients undergoing hemodialysis, patients with calcific uremic arteriolopathy, patients with atrial fibrillation, and patients with aortic valve stenosis, in order to investigate the reliability of dp-ucMGP and protein induced by vitamin K absence II (PIVKA-II) as representatives of vitamin K status in these patient groups [8]. Furthermore, they assessed the effect of vitamin K antagonist use as well as vitamin K supplementation on vitamin K status and the two aforementioned markers. The results of their study further push the boundaries and challenge available evidence regarding the role of vitamin K in the pathogenesis of vascular calcifications.

Considering the available data on vitamin K implications in arterial calcifications and atherogenesis, the area of research on vitamin K status in chronic kidney disease (CKD) is progressively expanding, and the promise of vitamin K supplementation in CKD is significant. Roumeliotis et al. provided a thorough review of the available evidence on vitamin K supplementation in uremic patients [9]. They cover in great detail the pathophysiology of vascular calcifications in CKD, highlighting relevant implications of vitamin K-dependent proteins in this process. They further present available clinical evidence linking vitamin K status with arterial calcification and adverse outcomes in patients within the whole spectrum of CKD. Next, to present the results of available studies on the effects vitamin K supplementation in patients with CKD, the authors address essential controversial vitamin K-related issues that clinicians may have to encounter in real-life clinical practice. Notably, the study by Rapp et al. showed that vascular vitamin K stores were diminished in dialysis patients, thus accelerating the process of vascular calcifications [8].

Finally, Kremer and coworkers examined the relationship between markers of vitamin K status, such as dephosphorylated uncarboxylated matrix Gla protein (dp-ucMGP) and uc osteocalcin (OC) with kidney function in a cohort of kidney transplant recipients and compared changes in plasma dp-ucMGP before and after kidney transplantation [10]. Taking into consideration the relative paucity of data examining the effect of kidney function on vitamin K-status parameters, the findings of this study further emphasize the fact that adequate adjustment for kidney function, or the use of kidney function-independent markers, such as proportions of uncarboxylated matrix Gla protein or osteocalcin, should be applied in patients with CKD so as to correctly estimate vitamin K status.

## 3. Conclusions

Ongoing research regarding Vitamin K and its derivatives is continuously trying to shed light on its molecular pathways of action, including a wide range of effects not related to coagulation. Although available evidence indicates promising beneficial effects with clinical implications especially with regard to cardiovascular disease and CKD specifically, pending questions remain regarding differences between different forms of Vitamin K and their therapeutic clinical utilization.

## References

[1] Kaneki M., Hosoi T., Ouchi Y., Orimo H. (2006). Pleiotropic actions of vitamin K: Protector of bone health and beyond?. Nutrition.

[2] Dam H. (1935). The antihemorrhagic vitamin of the chick. Occurrence and chemical nature. Nature.

[3] Halder M., Petsophonsakul P., Akbulut A.C., Pavlic A., Bohan F., Anderson E., Maresz K., Kramann R., Schurgers L. (2019). Vitamin K: Double Bonds beyond Coagulation Insights into Differences between Vitamin K1 and K2 in Health and Disease. Int. J. Mol. Sci..

[4] Furie B., Bouchard B.A., Furie B.C. (1999). Vitamin K-dependent biosynthesis of gamma-carboxyglutamic acid. Blood.

[5] Cozzolino M., Fusaro M., Ciceri P., Gasperoni L., Cianciolo G. (2019). The Role of Vitamin K in Vascular Calcification. Adv. Chronic Kidney Dis..

[6] Dahlberg S., Schött U., Eriksson E., Tahirsylaj Y., Schurgers L., Kander T. (2021). Intravenous Vitamin K1 for the Correction of Prolonged Prothrombin Times in Non-Bleeding Critically Ill Patients: A Prospective Observational Study. Nutrients.

[7] Schött U., Augustsson C., Lilover L., Nilsson C.U., Walther-Sturesson L., Kander T. (2021). Vitamin K Effects on Gas6 and Soluble Axl Receptors in Intensive Care Patients: An Observational Screening Study. Nutrients.

[8] Rapp N., Brandenburg V.M., Kaesler N., Bakker S.J.L., Stöhr R., Schuh A., Evenepoel P., Schurgers L.J. (2021). Hepatic and Vascular Vitamin K Status in Patients with High Cardiovascular Risk. Nutrients.

[9] Roumeliotis S., Duni A., Vaios V., Kitsos A., Liakopoulos V., Dounousi E. (2022). Vitamin K Supplementation for Prevention of Vascular Calcification in Chronic Kidney Disease Patients: Are We There Yet?. Nutrients.

[10] Kremer D., Groothof D., Keyzer C.A., Eelderink C., Knobbe T.J., Post A., van Londen M., Eisenga M.F., Schurgers L.J., TransplantLines Investigators (2021). Kidney Function-Dependence of Vitamin K-Status Parameters: Results from the TransplantLines Biobank and Cohort Studies. Nutrients.

